# Fluctuation of the Water Environmental Carrying Capacity in a Huge River-Connected Lake

**DOI:** 10.3390/ijerph120403564

**Published:** 2015-03-30

**Authors:** Hua Wang, Yiyi Zhou, Yang Tang, Mengan Wu, Yanqing Deng

**Affiliations:** 1 Key Laboratory of Integrated Regulation and Resource Development on Shallow Lake of Ministry of Education, College of Environment, Hohai University, Nanjing 210098, China; 2 College of Environment, Hohai University, Nanjing 210098, China; E-Mails: zyy664377643@163.com (Y.Z.); hhutangyang@163.com (Y.T.); wumengan@126.com (M.W.); 3 Hydrology Bureau of Jiangxi Province, Nanchang 330002, China; E-Mail: jxshuizhi@163.com

**Keywords:** Poyang Lake, river-connected lake, water environmental carrying capacity, numerical simulation, non-fully mixed coefficient, pollutant degradation coefficient

## Abstract

A new method, with the non-fully mixed coefficient (NFMC) considered, was put forward to calculate the water environmental carrying capacity (WECC) for huge river-connected lakes, of which the hydrological conditions always vary widely during a year. Poyang Lake, the most typical river-connected lake and the largest freshwater lake in China, was selected as the research area. Based on field investigations and numerical simulation, the monthly pollutant degradation coefficients and non-fully mixed coefficients of different lake regions were determined to explore the WECCs of COD, TN and TP of Poyang Lake in a common water year. It was found that under the hydrological conditions of a common water year the total WECCs of COD, TN and TP in the lake were respectively 181.9 × 10^4^ t, 33.3 × 10^4^ t and 1.86 × 10^4^ t. Due to the varied lake water volume and self-purification ability, an evident temporal fluctuation of WECCs in Poyang Lake was observed. The dry seasons were characterized by a higher NFMCs but lower WECCs owing to the lower water level and degradation ability. Variation coefficients of COD and TN WECC were close to each other, of which the average level was about 58.5%, a little higher than that of TP.

## 1. Introduction

Water environmental carrying capacity (WECC) is the maximum allowed pollutant load of a certain water body under the given hydrological conditions and water quality goals. Accurate evaluation of WECC values is of great significance to water environment protection and the maintenance of healthy aquatic ecosystems [1,2,3]. The terms “Assimilative Capacity” [4,5] and “Total Maximum Daily Loads” [6,7,8,9], *etc.*, used in Europe, also play the similar role in water environmental protection as WECC. Recently, many experts have carried out a series of related researches about rivers, lakes, reservoirs and coastal waters, and developed many WECC calculation methods for different water bodies [10,11,12]. However, little attention has been paid to the WECC calculation for river-connected lakes, of which the environmental characters always markedly differ from that of an isolated lake because of their special location.

In this kind of lakes, the frequent water volume exchange with external rivers usually makes it more difficult to estimate the WECCs in a fluctuating condition situation. Poyang Lake, the largest freshwater lake in China, is also the most typical river-connected lake at the middle and lower reaches of the Yangtze River, which plays an important role in maintaining regional ecological safety. To protect its water environment, a lot of work has been done [13,14,15], but research focused on WECC of the lake is still rare. A few scholars have conducted some initial studies, but they haven’t considered the fluctuation induced by the evident hydrological variation in the lake [16,17]. The results were far away from the actual capacities and were not suitable for guiding any practical water environmental protection work [18]. Therefore, in this paper, we selected Poyang Lake as the study area. The non-fully mixed coefficient (NFMC) was proposed to develop an easily-manipulated method for WECC calculation in a river-connected lake. The hydrological conditions in a common-water year 2010 was selected for typical study. By field investigation and numerical simulation, the pollutant degradation coefficients and non-fully mixed coefficients of different regions in each month were determined and the WECCs of COD, TN and TP of Poyang Lake in a common water year were quantitatively analyzed.

## 2. Materials and Methods

### 2.1. Study Area

**Figure 1:**
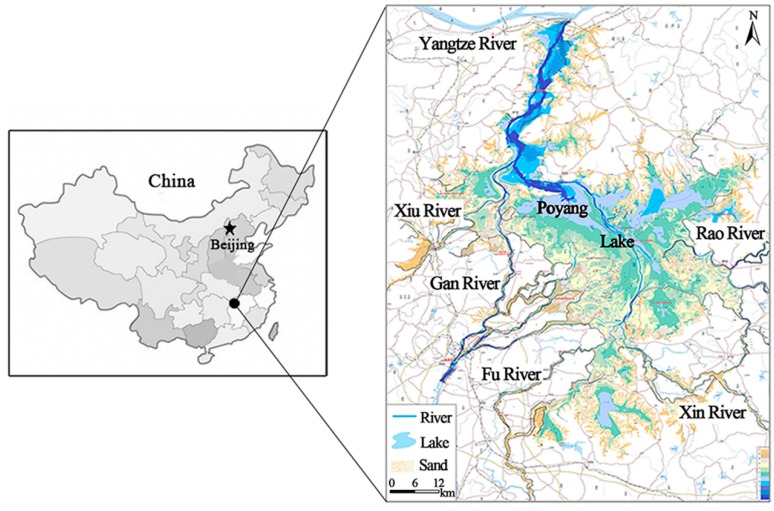
Location of the research area.

Poyang Lake (28°25'–29°45' N, 115°50'–116°44' E) is located on the south bank of the middle-lower Yangtze River in Jiangxi Province, China (Figure 1). The lake covers a series of districts including Nanchang, Xinjian, Jinxian, Yugan, Boyang, Duchang, Hukou, Jiujiang, Xingzi, De’an and Yongxiu. Poyang Lake is a typical river-connected lake. It regulates the water volume flowing from the five main upper rivers, namely the Gan River, Fu River, Xin River, Rao River and Xiu River, into the downstream Yangtze River. The lake can be divided into a south and a north part. The north is a waterway-like lake connecting the external Yangtze River, of which the average size is 40 km in length, 3–5 km in width, and 2.8 km at its narrowest. The south is the main lake, of which the size is 133 km in length and 74 km at its widest. Influenced by the external rivers, both the water volume and water surface area in the lake vary evidently in a year. The multi-year average water level of the lake is about 13.30 m, and the corresponding water area and volume are 2291.9 km^2^ and 2.1 × 10^9^ m^3^ respectively. The average annual rainfall in Poyang Lake is about 1632 mm, 74.4% of which is mainly concentrated between March and August. Water volume from the upstream five rivers and the internal runoff along the lake boundary are the main water supply sources for the lake. The average amount of water volume transported from the upstream five rivers to the lake is about 1.25 × 10^11^ m^3^ in a year, accounting for 87.1% of the total. Before 2003, the field measured data indicated that the water quality in the lake could reach category III of the National Surface Water Environmental Quality Standards in China. However, owing to the acceleration of regional industrialization and urbanization in recent years, Poyang Lake is faced with a lot of environmental problems. The increased nutrient input has caused significant water quality deterioration and eutrophication to some extent. The field investigation data from 2009 to 2010 showed that the water quality in most areas of the lake had decreased to category IV of the national standard, so it is very essential to accurately estimate its water environment capacity for the protection of the lake water environment.

### 2.2. WECC Calculation Methodology

The complicated water exchange among the upstream rivers, Poyang Lake, and the downstream Yangtze River results in a remarkable fluctuation of WECC in the lake. In addition, water pollutants are mainly transported into Poyang Lake by rivers and it should be considered that the broad lake morphology and the locations where the external rivers are related to the lake make it impossible for the input pollutants to fully mix with the whole lake. They can just be shaped as a mixing zone near the inflow area after being transported into the lake by the rivers. In view of this point, we proposed the non-fully mixed coefficient (NFMC) to optimize the present WECC calculation formula, and in this equation, the uneven distribution of pollutants under the hydrodynamic conditions in different months and districts are considered to avoid the overestimation or underestimation on WECC of the whole lake. The evolved calculation formula was written as in Equation (1):

(1) $$W_{ij}=\sum_{k=1}^{n}Q_{ik}\cdot (C_{sj}-C_{ij}^{k})+\sum_{l=1}^{m}\alpha_{i}^{l}K_{ij}^{l}V_{i}^{l}C_{sj}+Q_{ir}\cdot (C_{sj}-C_{ij}^{y})+\Delta w_{ij}$$

where $W_{ij}$ is the carrying capacity of factor *j* in month *i*; *m* and *n* are the number of districts of Poyang Lake and the number of inflowing rivers to the lake, respectively; $Q_{ik}$ is the water volume flowing into the lake from river *k* in month *i*; $C_{ij}^{k}$ is the average concentration of factor *j* in river *k* in month *i*; $C_{sj}$ is the water quality controlling standard of factor *j*, which is always determined by the local government with consideration of the socio-economic activities; $\alpha_{i}^{l}$ is the NFMC of district *l* in month *i*; $K_{ij}^{l}$ is the degradation coefficient of factor *j* in month *i* at district *l*; $V_{i}^{l}$ is the average storage capacity of district *l* in month *i*; $Q_{ir}$ is the water amount flowing from the Yangtze River backward into the Poyang Lake in month *i*; $C_{ij}^{y}$ is the concentration of factor *j* of the backward inflowing water in month *i*; $\Delta W_{ij}$ is the revised value of factor *j* in month *i*, and it is used to amend the influence of the non-generalized small rivers and rainfall on WECC. According to the monitoring data provided by Hydrology Bureau of Jiangxi Province, the water volume from the non-generalized small rivers and rainfall only accounts for 4% of the total. Their contribution to the lake WECC is far less than that of the generalized main rivers. So this item is calculated together as $\Delta w_{ij}\text{=}q_{i}(C_{sj}-\bar{c_{ij}})$ $\Delta w_{ij}\text{=}q_{i}(C_{sj}-\bar{c_{ij}})$ $W_{ij}=q_{i}\left(C_{sj}-\bar{c_{ij}}\right)$, where $q_{i}$ is the total water volume of the non-generalized small rivers and rainfall. $\bar{c_{ij}}$ is the average concentration of factor *j* of this part of water volume in month *i*.

### 2.3. Parameters Determination

#### 2.3.1. 2-D Unsteady Numerical Model

Here we improved the traditional 2-D unsteady water flow and quality model to simulate the pollutant transport processes between Poyang Lake and the external rivers. Combined with field investigations, tentative calculations were conducted to determine the pollutant degradation coefficients and NFMCs by month. The conservation forms of 2-D shallow water equations and the pollutant convection-diffusion equation can be written as in Equation (2) [19,20,21].

(2) $$\left\{ \begin{array}{l} \frac{\partial h}{\partial t}+\frac{\partial \left(hu\right)}{\partial x}+\frac{\partial \left(hv\right)}{\partial y}=0\\ \frac{\partial \left(hu\right)}{\partial t}+\frac{\partial \left(hu^{2}+gh^{2}/2\right)}{\partial x}+\frac{\partial \left(huv\right)}{\partial y}=gh\left(s_{0x}-s_{fx}\right)\\ \frac{\partial \left(hv\right)}{\partial t}+\frac{\partial \left(huv\right)}{\partial x}+\frac{\partial \left(hv^{2}+gh^{2}/2\right)}{\partial y}=gh\left(s_{0y}-s_{fy}\right)\\ \frac{\partial \left(hC\right)}{\partial t}+\frac{\partial \left(huC\right)}{\partial x}+\frac{\partial \left(hvC\right)}{\partial y}=\frac{\partial }{\partial x}\left(D_{x}h\frac{\partial C}{\partial x}\right)+\frac{\partial }{\partial y}\left(D_{y}h\frac{\partial C}{\partial y}\right)-KhC+S \end{array}\right.$$

where *h* is water depth; *t* is time; *u* and *v* are the depth-averaged velocity components in the *x* and *y* directions, respectively; *g* is acceleration of gravity; *S*_0*x*_ and *S_fx_* are the bed slope and friction slope in the *x* direction; *S*_0*y*_ and *S_fy_* are the bed slope and friction slope in the *y* direction; *D_x_* and *D_y_* are the dispersion coefficient of pollutants in the *x* and *y* directions under dynamic condition; *K* is the degradation coefficient; *C* is the depth-averaged pollutant concentration; *S* is the source-sink vector of pollutant. Due to the varied hydrodynamic conditions in Poyang Lake, the sediment pollutant release plays an important role in the lake water quality driving mechanism, so we supplement this item to enhance the simulation accuracy.

The water flow and water quality equations were combined to be calculated. Formulas (2) can be written as the unified form given in Equation (3) [22,23]:

(3) $$\frac{\partial q}{\partial t}+\frac{\partial f\left(q\right)}{\partial x}+\frac{\partial g\left(q\right)}{\partial y}=b\left(q\right)$$

where *q* is the vector of the conserved physical quantities; *f*(*q*) and *g*(*q*) are respectively the flux vectors in the *x* and *y* directions; *b*(*q*) is the source-sink vector; the detailed expressions are as follows: *q* = (*h*, *hu*, *hv*, *hC*)*^T^*; *f*(*q*) = (*hu, hu*^2^ + *gh*^2^/2, *huv*, *hC*)*^T^*; *g*(*q*) = (*hv, huv, hv*^2^ + *gh*^2^/2, *hC*)*^T^*; *b*(*q*)= (0, *gh*(*s*_0*x*_ ‒ *s_fx_*),*gh*(*s*0*y* − *sfy*),∇·(*D_i_*∇(*hC*)) ‒ *KhC* + *S*)*^T^*.

Developed in the framework of the finite volume method (FVM), the 2-D problem is transformed into a series of local 1-D Riemann problems, Detailed steps are documented in references [24,25]. To solve the Riemann equation, we compared the three high-performance numerical flux formulas: flux vector splitting (FVS) [26], flux difference splitting (FDS) [27], and the Osher scheme [28]. In this paper, we selected the FVS scheme, which has the merits of the finite difference method and finite element method, to improve the accuracy of the model.

#### 2.3.2. Confirmation of Degradation Coefficients

**Figure 2:**
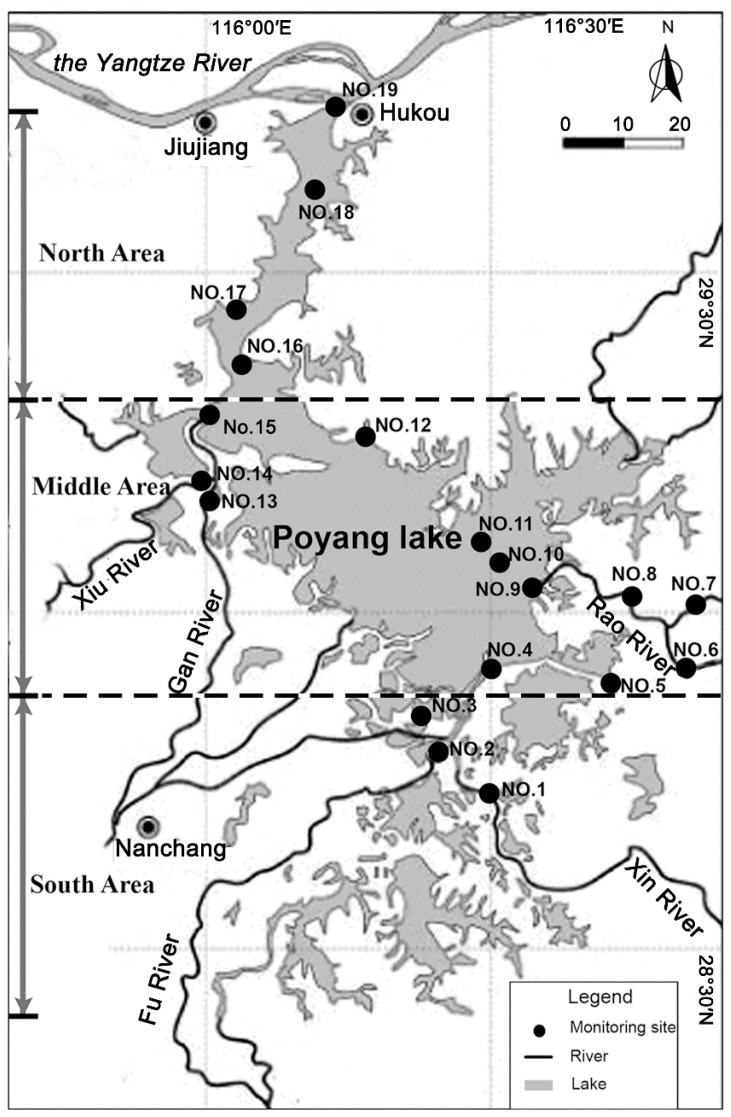
Field investigation sites in Poyang Lake.

Water quality degradation coefficients are always influenced by a combination of physical, chemical and biological factors. The uneven temporal-spatial distributions of hydrodynamic conditions and aquatic plants results in an evident seasonal and regional fluctuation of the degradation coefficients. Here, we combined the two methods of field investigation and numerical simulation to determine the detailed degradation parameters in Poyang Lake. Nineteen sites were set up in the lake for field water quality monitoring, with four points in the north area, 12 in the middle area and three in the south area (Figure 2). One year of consecutive monitored results collected in the common-water year 2010 was used as the sample data. By the established model the annual water environmental processes in Poyang Lake were simulated by month for more accurate results. The measured water quality concentrations and water levels of the upstream five rivers and the downstream Yangtze River were determined as the inflowing calculation boundaries, and the downstream Yangtze River was recognized as the outflowing calculation boundary. The input data of the above boundaries were supplied by the Hydrology Bureau of Jiangxi Province and the Bureau of Hydrology, Changjiang Water Resource Committee. Based on the topographic characteristics, the research area was divided into 7533 nodes and 6239 quadrilateral elements by Gambit and the average mesh size is about 700 m × 700 m. In view of the actual terrain characters, the roughness coefficients were arranged from 0.01 to 0.035. The wind drag coefficient and the horizontal eddy viscosity coefficient were respectively determined as 1.0 × 10^−3^ m^2^·s^−1^ and 0.5 × 10^5^ cm^2^·s^−1^. In the lake region, north winds were observed with the highest frequency, and during the simulation, the wind parameter was estimated as 3.01 m·s^−1^ [29,30], the average wind velocity at 10 m-elevation. To guarantee the calculation stability and accuracy, the time step was taken as 1 s. Under the given hydrological conditions and water quality input, tentative calculations were conducted to determine the pollutant degradation coefficients. When the calculated results at all field investigated points match well with the measured values (the average error is less than 10%), the optimized parameters in the model can be recognized as the integrated pollutant degradation coefficients in Poyang Lake. The indexes of COD, TN and TP were selected for research in this paper. Based on the calculated results, the degradation coefficients in a common-water year are shown in Table 1. The results indicated that the degradation abilities in Poyang Lake varied with time and location. The coefficients in the flood season (May–Oct.) were higher than those in the dry season (Nov.–Apr.). In the south and north areas of the lake, the degradation abilities were stronger than in the middle area due to the enhanced water volume exchanged with external rivers.

**Table 1:** Pollutant degradation coefficients of Poyang Lake in a common-water year.

Month	COD			TN			TP		
	North Area	Middle Area	South Area	North Area	Middle Area	South Area	North Area	Middle Area	South Area
Jan.	0.028	0.015	0.025	0.011	0.010	0.012	0.006	0.005	0.007
Feb.	0.031	0.017	0.028	0.012	0.011	0.013	0.007	0.006	0.008
Mar.	0.063	0.052	0.066	0.038	0.029	0.045	0.018	0.014	0.020
Apr.	0.081	0.072	0.087	0.053	0.040	0.064	0.025	0.019	0.026
May	0.088	0.080	0.095	0.059	0.044	0.070	0.027	0.021	0.029
Jun.	0.096	0.088	0.105	0.066	0.048	0.078	0.030	0.023	0.032
Jul.	0.102	0.087	0.104	0.065	0.048	0.077	0.030	0.023	0.032
Aug.	0.105	0.097	0.115	0.072	0.053	0.086	0.033	0.025	0.035
Sep.	0.100	0.092	0.109	0.068	0.050	0.082	0.031	0.024	0.033
Oct.	0.102	0.095	0.112	0.070	0.052	0.084	0.032	0.024	0.034
Nov.	0.066	0.055	0.069	0.041	0.031	0.048	0.019	0.015	0.021
Dec.	0.029	0.016	0.026	0.012	0.011	0.013	0.006	0.005	0.007

#### 2.3.3. Confirmation of Non-Uniform Distribution Coefficients

**Figure 3:**
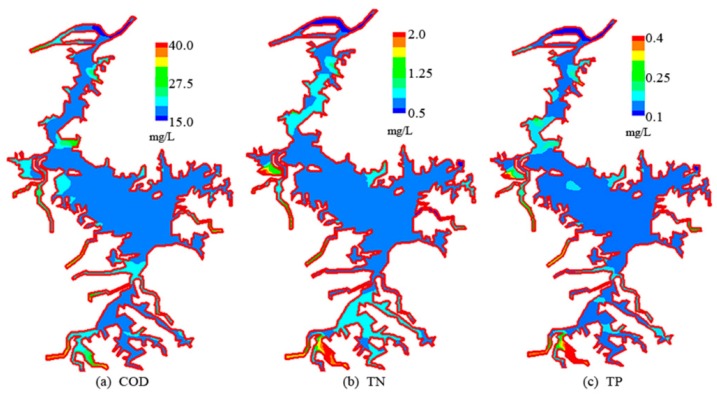
A typical distribution of pollutant in Poyang Lake in a common-water year.

The non-fully mixed coefficients reflect the degree of mixing of the pollutants with the lake water, which is closely related to hydrodynamic conditions, terrain characteristics and the water surface scale. In view of the complicated water volume exchange processes between the lake and the external rivers, we used the pollutant zone controlling method [31] to determine the NFMCs in Poyang Lake. The five upstream rivers—Gan River, Fu River, Xin River, Rao River and Xiu River—were considered the pollutant input boundaries and then we conducted tentative simulations under the same hydrological conditions by altering the pollutant input loads. Figure 3 provided the spatial distributions of COD, TN, and TP concentration during the flood season (Aug.) in 2010 as an example, which could visually reflect the uneven mixing of the pollutants in the lake.

Based on the massive calculated results, the total area of the pollutant zones for each calculation scheme was estimated against the same water quality control standard, and the curve of the relation between the input pollutant loads and mixing zone areas at each inflowing passage was established. When both the area of the pollutant zone at a certain input passage and that of all the pollutant zones in the lake could meet the control standard, the sum of the pollutants transported from all the input boundaries could be recognized as the maximum allowed pollutant load for the lakeshore area. In this paper we define the non-fully mixed coefficient as the ratio of this allowed pollutant load to the ideal carrying capacity of the lake, which was determined on the assumption that the pollutant transported into the lake was fully mixed with the water body. Specific formulas can be written as in Equation (4).

(4) $$\left\{ \begin{array}{l} \alpha_{ij}^{l}=\frac{\sum_{t=1}^{p}\max(W_{0ij}^{t})}{K_{ij}^{l}V_{i}^{l}C_{sj}}\\ f(W_{0ij}^{t})\leq f_{s},\sum_{l=1}^{m}\sum_{t=1}^{p}\max(W_{0ij}^{t})\leq f_{ts} \end{array}\right.$$

where $\alpha_{ij}^{l}$ is the non-fully mixed coefficient of index *j* at the j district in *i* month; *p* is the number of pollutant input passages in district *l*. $K_{ij}^{l}$ is the degradation coefficient of index *j* in month *i* at the district *l*; $V_{i}^{l}$ is the average storage capacity of district *j* in month *i*; *C_sj_* is the water quality control standards of index *j*. $W_{0ij}^{t}$ is the maximum input pollutant load of *j* index from the passage *t* in month *i*, and this item is determined by the pollutant zone control method. *f* is the correlation function between pollutant load and mixing zone area, which reflects the responding mechanisms of the pollutant zone area to the inputted pollutant load. Under the same hydrological conditions, the larger the pollutant loads are, the bigger the mixing zone will be. The function *f* is established by numerical simulation results. *f_s_* and *f_ts_* are respectively the area controlling standard for a single pollutant zone and the total zone area in the lake, which is always determined by the local government with the environmental and economic factors considered; During calculation, the zone area controlling standards *f_s_* and *f_ts_* were determined as in Equation (5), referring to the “Changing Mechanism of Water Quality in Poyang Lake” report, which has been finished in December of 2012 by the Hydrology Bureau of Jiangxi Province:

(5) $$\left\{ \begin{array}{l} f_{s}\leq 5km^{2}(D\leq 150m)\\ f_{s}\leq 7km^{2}(D>150m)\\ f_{ts}\leq A\times 2.5\% \end{array}\right.$$

where *D* is the width of the pollutant input passage; *A* is the lake area under a given water level. Based on the determined degradation coefficients, the NFMCs in Poyang Lake were calculated and are shown in Table 2. It was found that the NFMCs in Poyang Lake also varied with time and location. The flood seasons were characterized by the lower NFMCs, whereas those in the dry seasons were evidently increased. Influenced by the lake morphology, pollutants in the north and south parts were mixed more evenly than those in the middle region.

**Table 2:** Non-fully mixed coefficients of Poyang Lake in a common-water year.

Month	COD			TN			TP		
	North Area	Middle Area	South Area	North Area	Middle Area	South Area	North Area	Middle Area	South Area
Jan.	0.203	0.182	0.214	0.168	0.151	0.182	0.123	0.112	0.140
Feb.	0.134	0.120	0.141	0.111	0.099	0.120	0.081	0.074	0.092
Mar.	0.131	0.117	0.137	0.108	0.097	0.117	0.079	0.072	0.090
Apr.	0.133	0.119	0.140	0.110	0.099	0.119	0.080	0.073	0.092
May	0.063	0.057	0.067	0.052	0.047	0.057	0.038	0.035	0.044
Jun.	0.062	0.055	0.065	0.051	0.046	0.055	0.037	0.034	0.043
Jul.	0.059	0.053	0.062	0.048	0.043	0.053	0.035	0.032	0.040
Aug.	0.058	0.052	0.061	0.048	0.043	0.052	0.035	0.032	0.040
Sep.	0.065	0.058	0.068	0.054	0.048	0.058	0.039	0.036	0.045
Oct.	0.132	0.119	0.139	0.109	0.098	0.119	0.080	0.073	0.091
Nov.	0.140	0.119	0.140	0.110	0.099	0.119	0.080	0.073	0.092
Dec.	0.162	0.146	0.171	0.134	0.120	0.146	0.098	0.090	0.112

## 3. Results and Discussion

**Figure 4:**
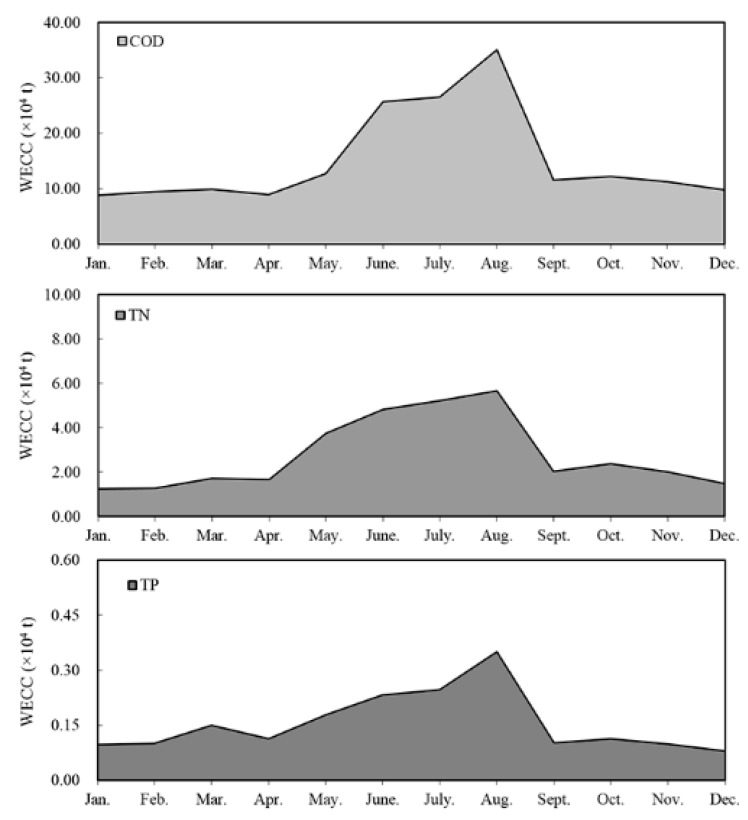
Fluctuation of WECC in Poyang Lake in a common-water year.

The hydrological conditions in the common water year 2010 were selected for evaluation. The monthly storage capacities of each district were estimated based on the field investigated water levels and the level-storage curve of the lake. According to the “Jiangxi Province Water Environmental Function Zoning”, the water quality controlling standard for the lake was set as grade III of the National Surface Water Environmental Quality Standards in China. The targeted values of COD, TN and TP are respectively 20 mg/L, 1.0 mg/L and 0.2 mg/L. With the determined water quality degradation coefficients and non-fully mixed coefficients, the WECC of Poyang Lake in a common-water year was calculated month by month. We explored the results of WECC to month-scale, because the hydrological conditions in Poyang Lake are relatively stable within a month and the results on this time scale are more useful for the pollutant control practiced by the local government. It was found that the total carrying capacities of COD, TN and TP in a common-water year were respectively 181.9 × 10^4^ t, 33.3 × 10^4^ t and 1.86 × 10^4^ t, and that they varied significantly within a year. During the flood seasons, the higher water level always results in a lower non-fully mixed coefficient, yet the huge water volume and strong self-purification ability still increased the WECC in the lake. The total WECCs of COD, TN and TP from May to October could separately reach 123.8 × 10^4^ t, 23.9 × 10^4^ t and 1.22 × 10^4^ t, which approximately accounted for 68.1%, 71.7% and 65.6% of that in the whole year. In the dry seasons, the WECC of the lake was markedly decreased due to the reduced water volume and weakened self-purification ability. The WECCs of COD, TN and TP from November to April were just 58.1 × 10^4^ t, 9.41 × 10^4^ t and 0.64 × 10^4^ t, accounting for only 31.5% of the total on average. In terms of the monthly WECC, the capacity values of COD, TN and TP all reached their maxima in August, which were 35.1 × 10^4^ t, 5.67 × 10^4^ t and 0.35 × 10^4^ t, whereas December and January were characterized by the lowest WECC. In these two months, the pollutants transported into the lake could be mixed to a higher extent due to the reduced water volume, but the poor dynamic conditions and the indigenous aquatic plants reduced the WECC. The average WECC level for these two months was just 23.4% of that in August. May and October could basically reflect the average monthly WECC values in a common-water year, and the WECCs of COD, TN and TP were respectively 12.45 × 10^4^ t, 3.07 × 10^4^ t and 0.15 × 10^4^ t on average. WECCs of COD, TN and TP in the lake fluctuated similarly within the year, yet the variation ranges differed. The variation coefficients of COD and TN WECC were close to each other, with an average level of about 58.5%, a little higher than that of TP (52.9%). Detailed results are shown in Figure 4. We applied the pollutant load data and the monitored water quality data from the “Changing Mechanism of Water Quality in Poyang Lake” report to validate the WECC results. It was observed that water qualities in the months when the pollutant load is lower than the WECC were overall better than that of the months when the WECC was less than the pollutant load, so the results match the actual capacities of the lake.

Poyang Lake is one of the World's six large-scale wetlands, with an extremely important ecological function. There are a series of national nature preserve areas and centralized drinking water source areas. The water quality of the lake is very essential to maintain a healthy ecosystem in these protected areas, and is closely related to the health of the people relying on the drinking water from the lake. WECC and the pollutant load on the lake are the two dominant factors driving the lake water quality. Here, we put forward a “Health Index” to present a further analysis, which can be written as in Equation (6):

(6) λi=PtiWECCi

where $\lambda_{i}$ is the health index of the lake in month *i*; $P_{ti}$ is the total pollutant load on the lake in month *i*; $WECC_{i}$ is the total water environment carrying capacity of the lake in month *i*.

**Figure 5:**
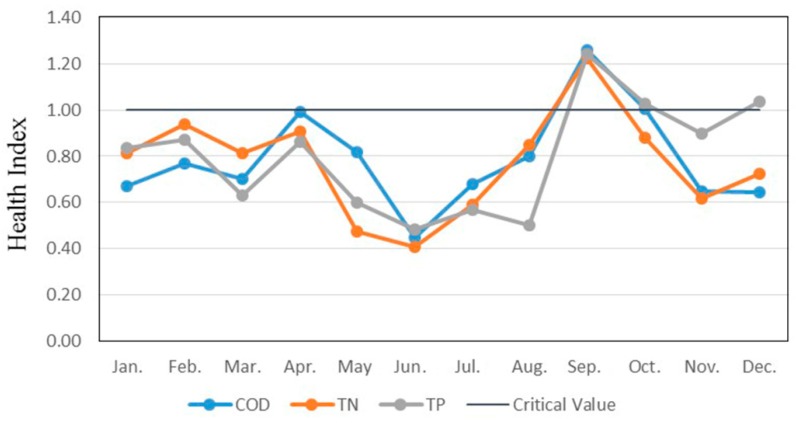
Health Index Processes in Poyang Lake.

According to the WECC results and the corresponding pollutant load in the common water year 2010, the health indexes for each month were calculated and are shown in Figure 5. It was found that: (1) The annual average health indexes of COD, TN and TP were respectively 0.79, 0.77 and 0.80; they were all lower than the critical value 1, which demonstrated that the WECC of Poyang lake could basically carry the present pollutant load and the drinking water quality and human health could be guaranteed overall. (2) September was characterized by the highest Health Index with an average value of approximately 1.24. The reason for this was the combination of the decreased carrying capacity and relatively higher pollutant load. (3) During the last four months in the year TP was observed to represent an evident higher health index, with an average level of 1.05. Efforts should be made in this period to control TP to maintain good water quality and human health.

Unlike an isolated lake, Poyang Lake is characterized by more complicated environmental characteristics due to its frequent water exchange with its external rivers. In this paper we aimed to establish an easily-manipulated method to calculate its WECC, with consideration of the temporal-spatial variation. The calculation equations developed in the paper have taken into account the combination of hydrodynamic conditions, water degradation capacity, and pollutant mixing processes, which is more reasonable to estimate the actual capacity. To maintain the water quality in a lake, WECC is a very important indicator against the pollutants from both the horizontal and vertical directions. It can be approximatively recognized as the maximum pollutant load on the lake, but it isn’t equal to the item of “maximum input pollutant load” used in Equation (4). The item is a theoretical value to limit the input load for a certain inflowing river under a given pollutant zone control standard. This item varies closely with hydrodynamic conditions and is just calculated by tentative simulation for the regional non-fully mixed coefficient determination to amend the item of *KVC_s_* in the equation. However, the above WECC results here aimed at the whole Poyang lake, which was implicitly composed of two main parts, the first one is the carrying capacity at the lakeshore area where the water quality is mainly dominated by the horizontal river input, and the second one is the carrying capacity in the offshore lake area where the effect of boundary input is remarkably reduced and the vertical atmospheric deposition and sediment release basically drive the water quality. The most important point we wanted to reveal in this paper is how to generate and concentrate these complicated calculation processes into a more easily-manipulated formula.

## 4. Conclusions

The most typical river-connected lake in China, Poyang Lake, was selected as the research area. Due to its special location and complicated water exchange with its external rivers, we proposed a non-fully mixed coefficient to optimize the present WECC calculation formula. Based on field investigations and numerical simulations, the pollutant degradation coefficients and non-fully mixed coefficients of different lake regions in each month were determined for Poyang Lake. The three factors of COD, TN and TP were selected for WECC evaluation by the new method. The results showed that the WECCs in Poyang Lake fluctuated evidently within a year. The high water level in the flood season results in a lower NFMC, but the huge water volume, good dynamic conditions and stable aquatic ecosystem contribute to a higher WECC. August is characterized by the peak WECC, whereas in January and December it was reduced to its lowest level. The COD, TN and TP WECCs fluctuated similarly in the year, yet their amplitudes were different. The WECC of TP was observed to show a relatively lower variation range than those of COD and TN. The results of this paper will play an important role in pollution control and water environmental protection for the study area, and will provide a reference for future in-depth studies on water environmental carrying capacity calculations in river-connected lakes.
